# Static and Dynamic Compressive Properties of Nano-Al_2_O_3_-Reinforced Epoxy Matrix Composites

**DOI:** 10.3390/polym18101228

**Published:** 2026-05-17

**Authors:** Jinzhu Li, Liwei Zhang, Jinchao Qiao

**Affiliations:** State Key Laboratory of Explosion Science and Safety Protection, Beijing Institute of Technology, Beijing 100081, China

**Keywords:** epoxy resin, nano-alumina, composite materials, Split Hopkinson pressure bar, strain rate effect, compressive properties, damage mechanism, impact resistance

## Abstract

This study investigates the influence of nano-alumina (nano-Al_2_O_3_) on the compressive properties and damage mechanisms of epoxy matrix composites across a wide strain rate range. Composites with varying nano-Al_2_O_3_ contents (0, 1, 3, 5, 10, 15 wt%) were tested under quasi-static (0.001~0.1 s^−1^) and dynamic (2500~4800 s^−1^) conditions using a universal testing machine and a Split Hopkinson Pressure Bar, respectively. The phase, the microstructure, and their effects on macro-mechanical performance and micro-damage were characterized by XRD, SEM, and TEM. Results indicate that the incorporated nano-Al_2_O_3_ is highly crystalline, single-phase lamellar α-Al_2_O_3_. Its addition significantly modulates the compressive properties, with effects dependent on both content and strain rate. Under quasi-static compression, yield strength increased monotonically with nano-Al_2_O_3_ content at 0.1 and 0.01 s^−1^, reaching a maximum increase of ~9.5% at 15 wt%. However, at 0.001 s^−1^, optimal strength occurred at 10 wt%, beyond which agglomeration caused degradation. Dynamic tests revealed a positive strain rate effect. The 10 wt% composite exhibited optimal overall performance, combining high peak stress and a stable stress plateau, whereas the 15 wt% sample showed higher peak stress but poor post-peak load-bearing capacity. Microstructural analysis showed that 10 wt% nano-Al_2_O_3_ dispersed uniformly, enhancing toughness by inhibiting crack propagation via interfacial bonding and microstructural refinement. In contrast, at 15 wt%, particle agglomeration induced interfacial defects, promoting debonding and brittle fracture. This work provides insights into the wide-strain-rate mechanical behavior of nanoparticle-reinforced polymers and supports the design of high-performance, impact-resistant epoxy composites.

## 1. Introduction

Epoxy resin (EP) is a core matrix for high-performance composites, and understanding its mechanical behavior is fundamental for material design and engineering applications. Epoxy resins are a class of thermosetting polymers renowned for their excellent mechanical properties (high strength and modulus), outstanding adhesion to various substrates, good chemical and corrosion resistance, low shrinkage during curing, and high electrical insulation [1]. These superior characteristics make them indispensable as matrix materials in advanced composites for a wide range of demanding applications, including aerospace [2], automotive [3], marine [4], construction [5], and electronic industries [6]. Research in this field has evolved from studying quasi-static loading to encompass complex scenarios involving multi-physical coupling, such as dynamic impact [7,8,9,10] and interfacial debonding [11,12,13,14]. For instance, Zhao et al. [15] systematically investigated the rate-dependent properties of epoxy under quasi-static tension and compression (strain rates: 0.001~0.1 s^−1^), revealing significant strain rate effects and a typical tension-compression asymmetry in elastic modulus, yield strength, and plastic flow.

To explore performance under extreme conditions, Bhagoria et al. employed Split/Modified Hopkinson Pressure Bars (SHPB/MHPB) with a low-temperature chamber [16]. They tested a bisphenol-A-based epoxy under dynamic compression (710~2051 s^−1^, −120 to −25 °C) and tension (1125~2194 s^−1^, −70 to −40 °C), finding that lower temperatures markedly increase strength and stiffness, with greater enhancement in compression.

The transition in failure mechanisms with loading rate has been examined microscopically. Wu et al. compared fracture surfaces after quasi-static and dynamic compression via SEM [17]. Quasi-static failure showed rough surfaces with plastic deformation and tortuous cracks, indicating ductile fracture. In contrast, dynamic impact produced smoother surfaces with river patterns, signifying brittle fracture. This shift is attributed to insufficient time for polymer chain mobility under high-rate loading, which inhibits plastic flow, promotes rapid stress concentration, and triggers instantaneous brittle failure. These findings collectively underscore the high strain-rate sensitivity of epoxy and its loading-dependent failure mechanisms.

To meet rising engineering demands, incorporating nanomaterials (e.g., nano-silica [18,19,20], graphene [21,22,23], carbon nanotubes [24,25,26], nano-rubber particles [27,28,29], Halloysite nanotubes (HNTs) [30,31,32]) into epoxy has become a key strategy for enhancing mechanical properties, toughness, and interfacial bonding, leveraging their high specific surface area and surface effects.

Dynamic mechanical properties under high strain-rate impact (10^2^~10^4^ s^−1^) are critical for applications in aerospace and defense. The SHPB technique is central to this research [33,34]. Studies show that epoxy nanocomposites generally exhibit a positive strain-rate effect, with dynamic modulus and strength increasing significantly with rate [35,36,37,38]. For example, graphene nanosheets can improve dynamic performance by approximately 18.5% at ~2300 s^−1^ [34]. However, this enhancement highly depends on filler type, content, and dispersion. Excessive or poorly dispersed filler (e.g., 5 wt% carbon black) can cause agglomeration, degrading energy absorption and dynamic properties.

A case in point is the work of Chihi et al. [36]. They incorporated multi-walled carbon nanotubes (0, 0.5, 2 wt%) into an epoxy/carbon fiber composite and conducted SHPB tests at varying pressures, using high-speed cameras to record damage evolution. Results showed that carbon nanotubes significantly enhanced dynamic properties and fracture resistance by reinforcing the matrix and improving fiber-matrix bonding. High-speed footage confirmed that increased nanotube content reduced macroscopic damage, delamination, and crack propagation, with the 2 wt% specimen showing no visible damage at 1.8 bar pressure. This confirms that well-dispersed nanofillers can effectively improve the dynamic response and damage tolerance of epoxy composites.

Owing to their excellent properties, epoxy composites are widely used in load-bearing and protective structures in aerospace [39,40], defense [41,42], and rail transportation [43,44]. As technology advances toward higher speeds and more extreme environments, understanding material response and failure mechanisms across a wide strain-rate range—particularly under high-speed impact—has become a key scientific challenge. Reinforcing epoxy with nanoparticles is a vital strategy for performance enhancement. Among various nanofillers, nano-alumina (nano-Al_2_O_3_) is promising due to its high modulus, strength, and chemical stability [45,46,47]. Nevertheless, research on the mechanical properties of nano-Al_2_O_3_/epoxy composites across a wide strain-rate range remains insufficient. The dynamic mechanical behavior and damage mechanisms, in particular, are not well understood. Existing studies have yet to clarify the influence of key factors like nano-Al_2_O_3_ content and strain rate on composite strength and microscopic damage, limiting the performance optimization and engineering application of these materials.

Therefore, this study combines experimental and theoretical analysis to systematically investigate the compressive response and damage mechanisms of nano-Al_2_O_3_/epoxy composites from quasi-static to dynamic impact loading over a wide strain-rate range. The specific objectives are to: (1) analyze the effect of nano-Al_2_O_3_ content on the compressive mechanical behavior (strength, modulus, toughness) of epoxy composites under quasi-static and dynamic loading; and (2) explore the rate-dependent characteristics, failure modes, and microscopic damage evolution mechanisms of the composites within a strain-rate range of 10^−3^ to 10^3^ s^−1^. The findings are expected to deepen the understanding of the mechanical behavior and strengthening/toughening mechanisms of nanoparticle-reinforced polymers under wide strain-rate loading. From an engineering perspective, this work provides reliable experimental data and a theoretical basis for designing, optimizing, and assessing the safety of high-performance, impact-resistant epoxy composites.

## 2. Materials and Methods

### 2.1. Materials and Specimen Preparation

The epoxy adhesive used was DP460, a two-component toughened structural adhesive from 3M (Maplewood, MN, USA). The base agent (Component A) is a white paste, and the curing agent (Component B) is an amber liquid, mixed at a volume ratio of 2:1 (A:B). The cured adhesive is off-white. At 22 °C, it has a workable time of 60 min, develops handling strength in 4 h, and achieves initial cure in 24 h.

Nano-Al_2_O_3_ powder (purity 99.9%, particle size ≤500 nm) was used as the filler. Specimens with six different mass fractions were prepared: 0 wt%, 1 wt%, 3 wt%, 5 wt%, 10 wt%, and 15 wt%.

Based on the preparation process for nano-Al_2_O_3_-reinforced epoxy composites and the requirement for six specimens with different filler contents, specialized laboratory instruments and equipment were selected for each stage, covering the entire procedure from batching, dispersion, mixing, debubbling, curing, demolding, to cutting. This ensured experimental repeatability and the fabrication of qualified specimens meeting testing standards.

The following equipment was used:

Batching: An electronic balance (Lucky, Hyderabad, India, accuracy 0.01 g, range 0–1 kg), beakers, and glass stirring rods were used to accurately weigh the three raw materials: epoxy resin, nano-Al_2_O_3_, and curing agent, ensuring precise proportions for the six different formulations.

Dispersion: An ultrasonic cleaner (SUNNE, Hong Kong, China, ultrasonic power 0–700 W, frequency 28 kHz) was employed to achieve uniform dispersion of nano-Al_2_O_3_ within the epoxy matrix and to mitigate agglomeration.

Mixing: An electric stirrer (LICHEN, Xiamen, China, speed 0–1000 rpm) was used to thoroughly homogenize the dispersed nano-Al_2_O_3_/epoxy mixture with the curing agent.

Debubbling: A vacuum debubbling chamber (FUJIWARA, Osaka, Japan, vacuum range: −0.1–0 MPa) was utilized to remove entrapped air bubbles from the mixed system, preventing them from compromising the mechanical performance of the specimens.

Curing: A constant-temperature drying oven (LICHEN, Shaoxing, China, temperature range: 10–250 °C, set to 25 °C) and poly (ethylene terephthalate) glycol (PETG) molds were used for curing and shaping the specimens.

Demolding: Demolding tools, including pry bars and a hammer, were used to carefully remove the cured specimens from the molds without damage.

Cutting: A lathe and a milling machine were used to cut the demolded specimens to the dimensions required by testing standards, followed by surface and edge polishing to ensure dimensional accuracy.

**Figure 1:**
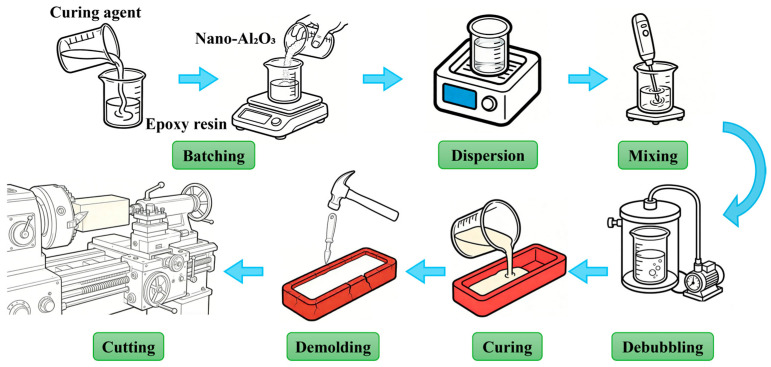
Flow chart of the preparation process for nano-Al_2_O_3_/epoxy composites.

The optimized direct dispersion process route adopted in this study is shown in Figure 1. The complete workflow began with accurate batching, followed by a multi-stage dispersion phase—“mechanical pre-stirring → ultrasonic dispersion → high-speed shear mixing”—which served as the core optimized step. This stage was designed to provide differentiated shear and cavitation energy input according to the different filler content gradients, specifically to address agglomeration challenges. Subsequently, defects were eliminated via vacuum debubbling, and the mixture was shaped under controlled curing conditions. Finally, standard specimens were obtained via machining. This design ensured process repeatability and adaptability to different filler contents.

Based on the optimized direct dispersion process route (Figure 1), the implementation encompassed key steps including batching, dispersion, mixing, debubbling, curing, and demolding. The specific operations and control requirements for each step are as follows:

(1) Batching

According to the designed nano-Al_2_O_3_ contents (0, 1, 3, 5, 10, 15 wt%), the required mass of each raw material was calculated and recorded in a batching sheet. Weighing was performed using an electronic balance with an accuracy of 0.01 g: the epoxy resin and curing agent were weighed at a fixed mass ratio of 2:1, and the nano-Al_2_O_3_ mass was calculated based on the corresponding weight fraction. The weighed epoxy resin, nano-Al_2_O_3_, and curing agent were placed separately in dry, clean beakers and clearly labeled. Spillage was avoided during weighing. Each batch formulation was weighed twice, with the error controlled within ±0.01 g. The raw material quantities, weighing time, and ambient temperature (25 °C) for each specimen were documented to form a complete batching record, ensuring experimental repeatability. Batches for the six different content specimens were prepared and stored separately to prevent confusion.

(2) Dispersion

To improve the dispersion of nanoparticles and mitigate their tendency to agglomerate in the direct dispersion process, a “stepwise” ultrasound–mechanical synergistic dispersion method was employed. The specific procedure was as follows: After slight manual pre-stirring of the weighed epoxy resin, the nano-Al_2_O_3_ powder was added, followed immediately by ultrasonic treatment. Considering the varying dispersion difficulty due to different filler contents, dispersion parameters were set differentially according to the content gradient (see Table 1). For low-content specimens (1 wt%), ultrasonic treatment was conducted at 250 W for 10 min. For medium- to high-content specimens (3–15 wt%), the power was increased to 300 W and the duration extended to 15 min to suppress particle agglomeration through higher energy input. During the process, manual stirring was performed every 5 min to dislodge agglomerates adhering to the container walls.

(3) Mixing

The dispersed slurry was stirred at 500 rpm for 5 min. The mixing process ensured thorough material flow. The container walls were scraped if necessary, and mixing resumed until the slurry appeared homogeneous without lumps or stratification. After mixing, the quality was inspected: a uniform, transparent or semi-transparent system with no visible particles or phase separation was deemed qualified. If stratification or agglomeration persisted, mixing was extended for an additional 5 min until uniformity was achieved.

(4) Debubbling

Bubbles entrapped during mixing can affect the compactness and mechanical properties of the specimens and therefore require removal. The uniformly mixed slurry was placed in a vacuum debubbling device and treated at a vacuum of −0.08 MPa for 5 min, until bubbles on the liquid surface largely disappeared and the surface became smooth. If the liquid level dropped after debubbling, a small amount of slurry was added to level it with the mold opening, ensuring the final specimen dimensions met requirements.

(5) Curing

Considering that the introduction of nanoparticles might affect the contact and reaction between the epoxy resin and curing agent, the curing process was controlled. The debubbled slurry was poured into molds, subjected to a second brief debubbling, and then cured under constant-temperature conditions at 25 °C. Curing time was set according to a gradient based on nano-Al_2_O_3_ content: 24 h for low-content systems, 36 h for medium-content, and extended to 48 h for high-content systems. This ensured the formation of a complete three-dimensional cross-linked network for all filler proportions, aiming to counteract potential poor interfacial bonding that might arise from the dispersion process.

(6) Demolding

Before demolding, specimens were checked for adhesion to the mold. If significant adhesion was observed, a small amount of anhydrous ethanol was applied to the mold edges and allowed to penetrate briefly to facilitate separation. Demolding tools were then used to gently pry the edges, allowing the specimen to be released intact, taking care not to scratch the specimen surface. After demolding, specimens were placed on a clean surface for visual inspection. Specimens with bubbles, cracks, insufficient filling, or obvious adhesion marks were discarded. Qualified specimens were marked according to their nano-Al_2_O_3_ content and stored separately.

(7) Cutting

The dimensions of the demolded specimen blanks were larger than the standard sizes required for subsequent mechanical property testing, necessitating cutting and polishing. Target specimen dimensions were determined based on the requirements for quasi-static and dynamic mechanical tests. Cutting was performed on a lathe along marked lines, with specimens securely fixed to avoid vibration-induced dimensional errors or edge damage. After cutting, fine sandpaper was used to polish the specimen surfaces and edges, removing burrs and cutting marks to achieve smooth, flat surfaces and edges, while avoiding over-polishing that could cause dimensional deviation. Finally, a vernier caliper was used to measure key specimen dimensions (height, diameter). Each dimension was measured at 3–5 different locations, and the average value was taken. Specimens failing to meet dimensional tolerances were reworked until all requirements were satisfied.

**Figure 2:**
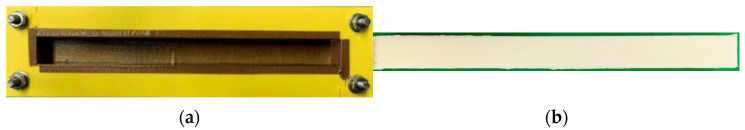
Preparation process for quasi-static compression specimens: (**a**) 3D-printed mold; (**b**) Cured rectangular specimen.

A mold was fabricated from polyethylene terephthalate-1,4-cyclohexanedimethanol (PETG) via Fused Deposition Modeling (FDM) 3D printing (Figure 2a). The uniformly mixed nano-Al_2_O_3_/epoxy slurry was poured into the mold and cured for 48 h. The resulting rectangular composite specimens were then demolded (Figure 2b).

**Figure 3:**
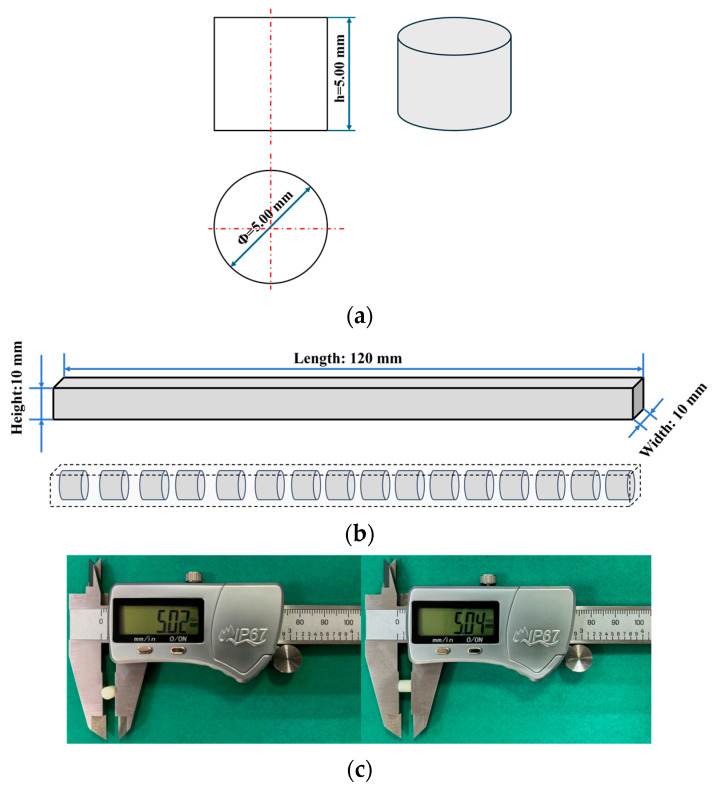
Schematic and photograph of the quasi-static compression specimens: (**a**) Dimension diagram; (**b**) Machining schematic; (**c**) Photograph of machined specimens.

Figure 3a shows the dimensional schematic for the compression specimens. The rectangular blanks (120 mm × 10 mm × 10 mm) were machined into cylindrical specimens with dimensions of Φ5.00 mm × 5.00 mm (Figure 3b). Specimens for all six filler contents were prepared identically. At least three valid specimens were prepared for each condition. Figure 3c shows a photograph of the machined specimens, with diameters and heights within the allowable tolerance.

### 2.2. Quasi-Static Compression Test

Quasi-static compressive tests of the nano-Al_2_O_3_-reinforced epoxy resin composites were conducted on an electronic universal testing machine. The machine, driven by a hydraulic system, applied compressive loading via a compression indenter. Key parameters, including loading time, indenter displacement, and applied load, were automatically recorded by the integrated signal acquisition system to ensure data accuracy and integrity.

The basic mechanical responses were calculated from the acquired signals. Engineering stress ($\sigma_{E}$) and engineering strain ($\varepsilon_{E}$) were determined using the following equations [48]:

(1) $$\sigma_{E}=\frac{F}{A_{0}}$$

(2) $$\varepsilon_{E}=\frac{L_{0}-L_{i}}{L_{0}}$$

where F is the measured load, $A_{0}$ and $L_{0}$ are the initial cross-sectional area and initial length of the specimen, and $L_{i}$ is the instantaneous length of the specimen gauge section. The nominal strain rate ($\dot{\varepsilon }$) is given by:

(3) $$\dot{\varepsilon }=\frac{\Delta \varepsilon }{\Delta t}$$

where Δε is the strain increment and Δt is the corresponding time increment.

The stress and strain calculated from Equations (1) and (2) are engineering values, which do not account for the change in the specimen’s geometric dimensions during deformation. To improve data accuracy, especially under large plastic deformation where significant changes in cross-sectional area and length occur, engineering values were converted to true stress ($\sigma_{T}$) and true strain ($\varepsilon_{T}$) as follows:

(4) $$\left\{\begin{array}{c} \sigma_{T}=\sigma_{E}\left(1+\varepsilon_{E}\right)\\ \varepsilon_{T}=\ln\left(1+\varepsilon_{E}\right) \end{array}\right.$$

where $\sigma_{E}$ and $\varepsilon_{E}$ are the engineering stress and engineering strain obtained from Equations (1) and (2), respectively.

### 2.3. Dynamic Compression Test

**Figure 4:**
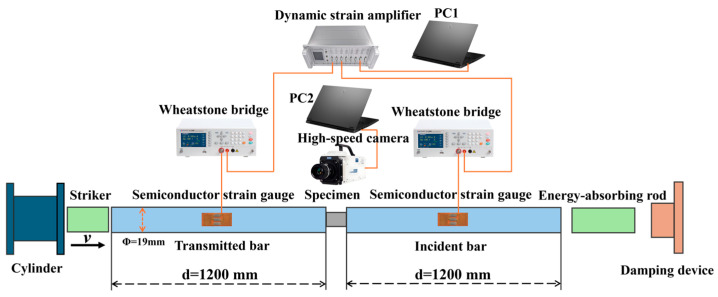
Schematic diagram of the SHPB experimental system.

Dynamic compressive tests were performed on an SHPB system, the schematic of which is shown in Figure 4. In the test, a striker bar launched by a gas gun impacts the incident bar, generating a one-dimensional compressive incident stress wave ($\varepsilon_{I}$). Upon reaching the bar-specimen interface, part of the wave is reflected ($\varepsilon_{R}$), and the remainder is transmitted ($\varepsilon_{T}$) through the specimen into the transmitted bar. Strain gauges mounted on the bars capture these wave signals, which are amplified and recorded for subsequent analysis of the specimen’s dynamic stress–strain response.

**Figure 5:**
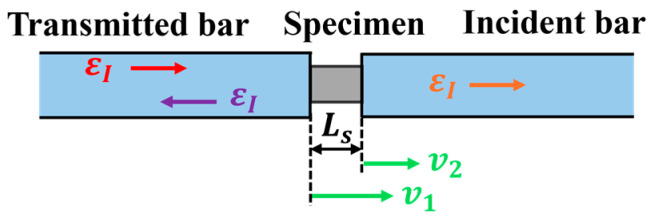
Schematic of incident wave, transmitted wave, and particle velocity in SHPB tests.

The data analysis is based on the assumptions of one-dimensional stress wave propagation and uniform stress–strain distribution within the specimen [49,50,51]. The particle velocities at the interfaces between the specimen and the incident bar ($v_{1}$) and the transmitted bar ($v_{2}$) are given by (see Figure 5):

(5) $$v_{1}=C_{B}\left(\varepsilon_{I}-\varepsilon_{R}\right)$$

(6) $$v_{2}=C_{B}\varepsilon_{T}$$

where $C_{B}$ is the longitudinal wave velocity in the bar, and $\varepsilon_{I}$, $\varepsilon_{R}$ and $\varepsilon_{T}$ are the strains corresponding to the incident, reflected, and transmitted waves, respectively.

The strain rate ($\dot{\varepsilon }$), strain (ε), and stresses at the two specimen ends ($\sigma_{1}$, $\sigma_{2}$) are derived as follows [49,50,51]:

(7) $$\dot{\varepsilon }=\frac{v_{1}-v_{2}}{L_{S}}=\frac{C_{B}}{L_{S}}\left(\varepsilon_{I}-\varepsilon_{R}-\varepsilon_{T}\right)$$

(8) $$\varepsilon =\int_{0}^{t}\dot{\varepsilon }dt=\frac{C_{B}}{L_{S}}\int_{0}^{t}\left(\varepsilon_{I}-\varepsilon_{R}-\varepsilon_{T}\right)dt$$

(9) $$\sigma_{1}=\frac{A_{B}}{A_{S}}E_{B}\left(\varepsilon_{1}+\varepsilon_{R}\right)$$

(10) $$\sigma_{2}=\frac{A_{B}}{A_{S}}E_{B}\varepsilon_{T}$$

where $L_{S}$ is the original specimen length, and $A_{B}$, $A_{S}$, and $E_{B}$ are the cross-sectional area of the bar, the cross-sectional area of the specimen, and the elastic modulus of the bar material, respectively.

Applying the stress equilibrium assumption ($\sigma_{1}=\sigma_{1}$) yields:

(11) $$\varepsilon_{I}+\varepsilon_{R}=\varepsilon_{T}$$

Substituting Equation (11) into Equations (7)–(9) leads to the simplified formulas commonly used for SHPB data processing:

(12) $$\dot{\varepsilon }=-2\frac{C_{B}}{L_{S}}\varepsilon_{R}$$

(13) $$\varepsilon =-2\frac{C_{B}}{L_{S}}\int_{0}^{t}\varepsilon_{R}dt$$

(14) $$\sigma =\frac{A_{B}}{A_{S}}E_{B}\varepsilon_{T}$$

where compression is defined as positive for strain rate and stress.

### 2.4. Test Matrix

Quasi-static compression tests were performed on specimens with all six filler contents (0–15 wt%) at three strain rates: 0.001 s^−1^, 0.01 s^−1^, and 0.1 s^−1^.

Dynamic compression tests using the SHPB were conducted on specimens with four selected filler contents: 0 wt%, 5 wt%, 10 wt%, and 15 wt%. The strain rate was controlled by adjusting the gas gun pressure to three levels: 0.10 MPa, 0.15 MPa, and 0.20 MPa. The complete test matrix is listed in Table 2.

## 3. Results and Discussion

### 3.1. Characterization of Nano-Al_2_O_3_ Powder and Its Composites

**Figure 6:**
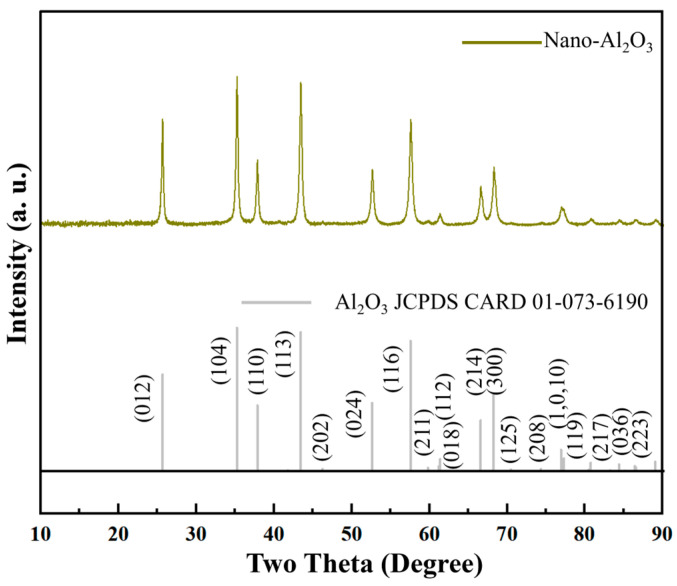
XRD pattern of the nano-Al_2_O_3_ powder.

X-ray Diffraction (XRD, BRUKER D8 advance) phase analysis of the nano-Al_2_O_3_ powder was conducted, with the pattern shown in Figure 6. All diffraction peaks match the standard pattern for rhombohedral α-Al_2_O_3_ (JCPDS Card No. 01-073-6190), confirming its high purity and single-phase composition. The main peaks at 2θ = 25.6°, 35.1°, and 43.4° correspond to the (012), (104), and (113) crystal planes, respectively, with the (104) plane showing the highest intensity.

**Figure 7:**
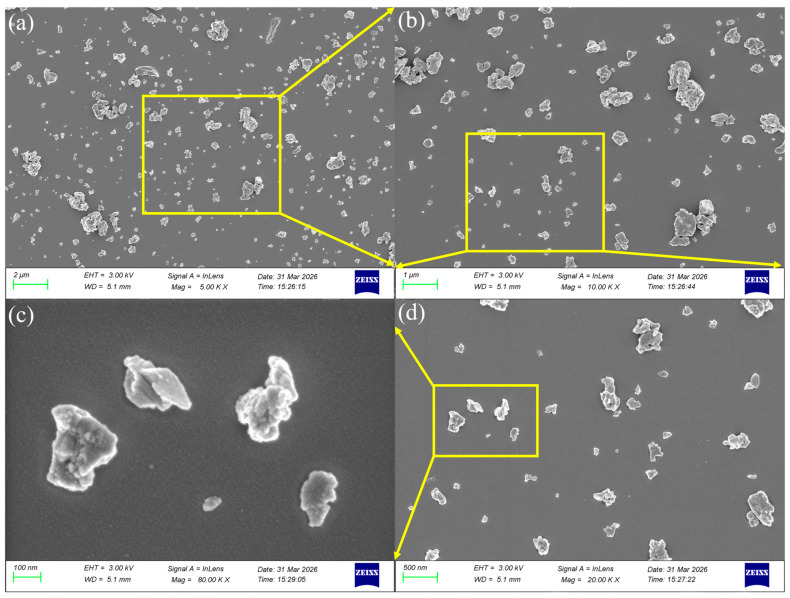
SEM images of nano-Al_2_O_3_: (**a**) 5000× magnification; (**b**) 10,000× magnification; (**c**) 80,000× magnification; (**d**) 20,000× magnification.

Scanning Electron Microscopy (SEM, ZEISS 300, Oberkochen, Germany) was used to characterize the powder’s morphology, as shown in Figure 7. The low-magnification image (Figure 7a, scale bar: 2 µm) and higher-magnification images (Figure 7b–d) reveal that the Al_2_O_3_ particles exhibit a distinct lamellar structure with planar dimensions mainly between 100 nm and 500 nm. This flake-like morphology is considered favorable for interfacial contact with the epoxy resin matrix.

**Figure 8:**
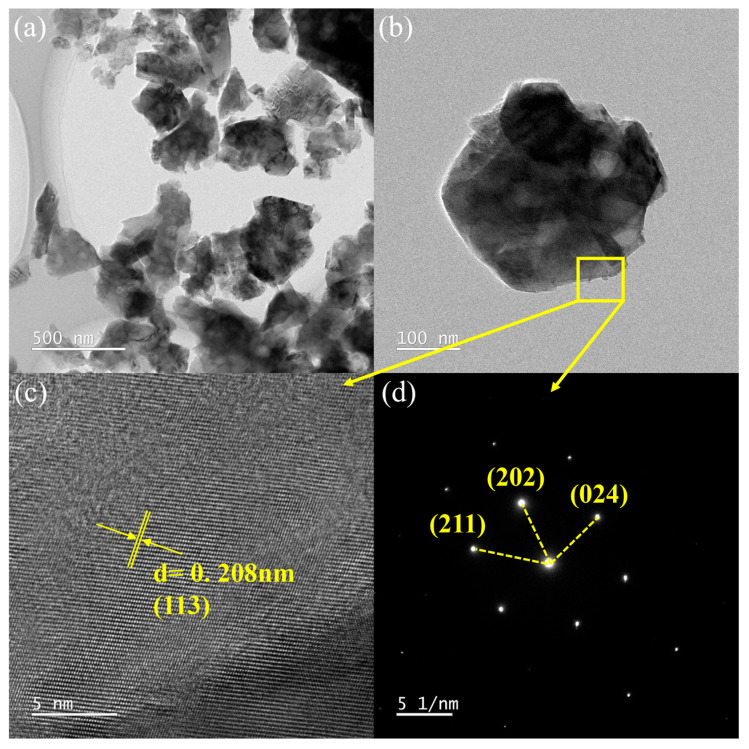
TEM characterization of nano-Al_2_O_3_: (**a**,**b**) Morphology; (**c**) HRTEM image showing lattice fringes; (**d**) SAED pattern.

Transmission Electron Microscopy (TEM, FEI-TALOS-F200X, Waltham, MA, USA) was further employed. Figure 8a (scale bar: 500 nm) confirms the lamellar morphology with a lateral size of approximately 500 nm. A local magnification (Figure 8b) shows a disk-like thin-plate structure. High-Resolution TEM (HRTEM) on the region marked in Figure 8b (Figure 8c) displays clear lattice fringes with an interplanar spacing of about 0.208 nm, matching the theoretical d-spacing of the (113) plane of α-Al_2_O_3_. The corresponding Selected Area Electron Diffraction (SAED) pattern (Figure 8d) shows sharp diffraction spots, confirming the single-crystal nature. These results are consistent with the XRD analysis, verifying the nano-Al_2_O_3_ as highly crystalline, single-phase α-Al_2_O_3_.

**Figure 9:**
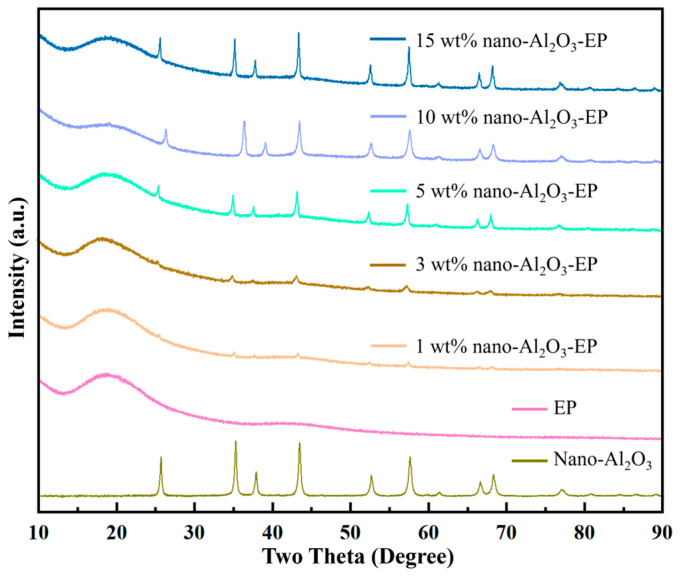
XRD patterns of epoxy composites with different nano-Al_2_O_3_ contents.

XRD analysis was performed on the fabricated composites. For the neat epoxy (0 wt%), a broad diffuse halo characteristic of an amorphous structure is observed (Figure 9). With the addition of nano-Al_2_O_3_, distinct crystalline diffraction peaks corresponding to α-Al_2_O_3_ appear superimposed on the amorphous background. The peak intensity increases and sharpens with higher filler content, becoming particularly prominent at 15 wt%, confirming the successful incorporation of crystalline nano-Al_2_O_3_ into the epoxy matrix.

Notably, the amorphous peak in the 10 wt% composite appears less pronounced than in the 15 wt% sample. This observation can be attributed to the filler’s dispersion state and its effect on the XRD peak profile. At 10 wt%, the nano-Al_2_O_3_ particles may reach an optimal dispersion with minimal agglomeration, leading to diffraction peaks that are not only more intense but also broader and more diffuse due to enhanced particle–matrix interaction and reduced crystalline coherence length. This broadened scattering signal superimposes over a wider 2θ range, effectively masking a larger portion of the amorphous background. In contrast, at 15 wt%, increased filler loading may induce slight agglomeration, resulting in sharper and more localized diffraction peaks. While these peaks are stronger in absolute intensity, their narrower profile leaves the amorphous halo more visually distinct in regions between the sharper crystalline reflections. Thus, the apparent reduction in the amorphous peak at 10 wt% likely results from a more homogeneously dispersed filler phase producing a broad, strong scattering signal that overshadows the epoxy matrix background more uniformly across the measured angular range.

### 3.2. Analysis of Quasi-Static Compression Experimental Results

**Figure 10:**
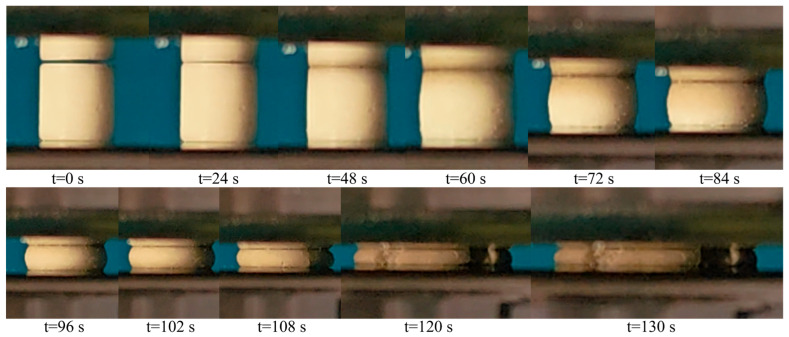
Quasi-static compression deformation process of a nano-Al_2_O_3_/epoxy composite at a strain rate of 0.01 s^−1^.

Figure 10 shows the deformation sequence of a nano-Al_2_O_3_/epoxy composite specimen during quasi-static compression at a strain rate of 0.01 s^−1^. Under axial loading, the specimen undergoes significant axial shortening and radial expansion. The radial expansion increases progressively from the loading end towards the midsection, ultimately resulting in a characteristic barrel-shaped deformation, as visible at t = 96 s and 108 s.

**Figure 11:**
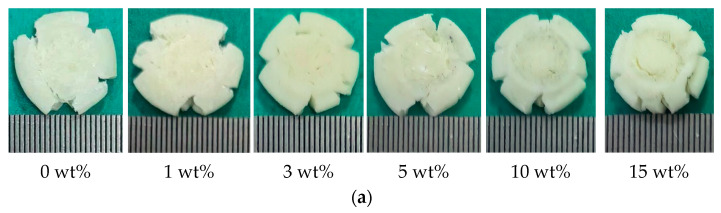

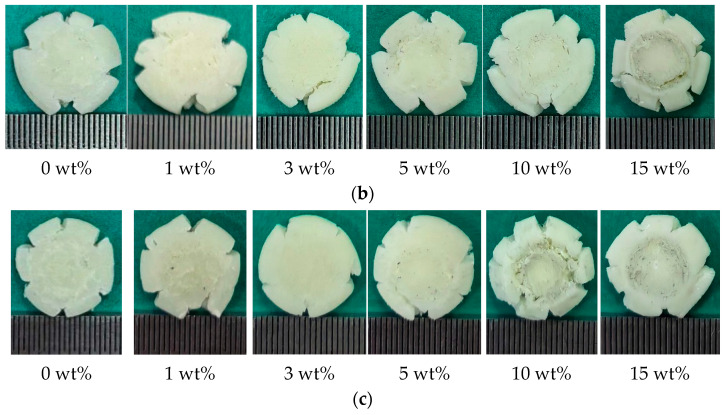
Compressive macroscopic damage morphology of epoxy composites with different nano-Al_2_O_3_ contents at strain rates of: (**a**) 0.1 s^−1^, (**b**) 0.01 s^−1^, and (**c**) 0.001 s^−1^.

A comparison of the post-failure macroscopic morphology for composites with different nano-Al_2_O_3_ contents at three quasi-static strain rates is shown in Figure 11. All specimens, initially cylindrical (Φ5.00 mm × 5.00 mm), exhibited obvious damage after testing. Numerous circumferential and axial cracks are present on the surfaces, resulting in a petal-like failure mode. Composites with different filler contents show similar macroscopic damage characteristics across the tested strain rates.

**Figure 12:**
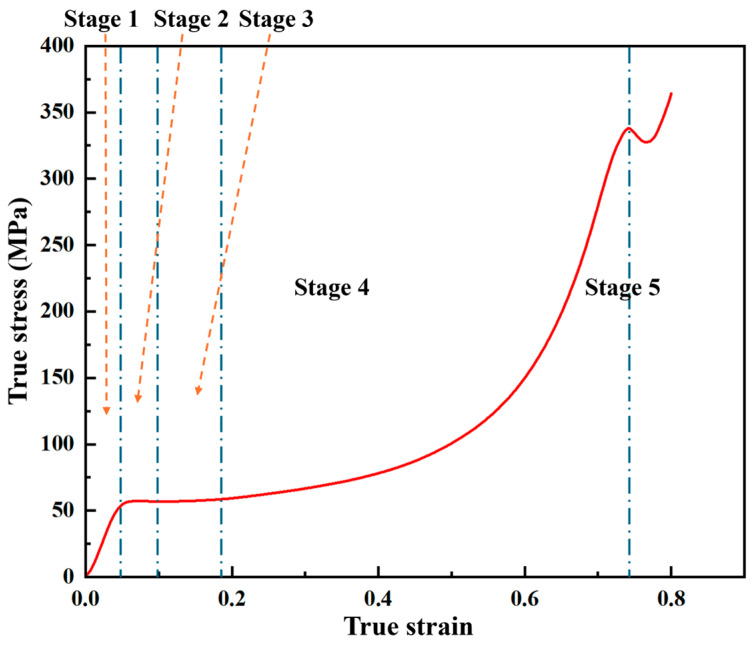
Typical deformation stages identified in the compressive stress–strain curve of the 5 wt% nano-Al_2_O_3_/epoxy composite.

The true stress-true strain curves for all composites exhibit five characteristic stages, as illustrated for the 5 wt% composite in Figure 12: (1) an initial linear-elastic stage, (2) a nonlinear yielding stage where stress peaks, (3) a strain-softening stage, (4) a subsequent strain-hardening stage, and (5) final fracture or unloading.

**Figure 13:**
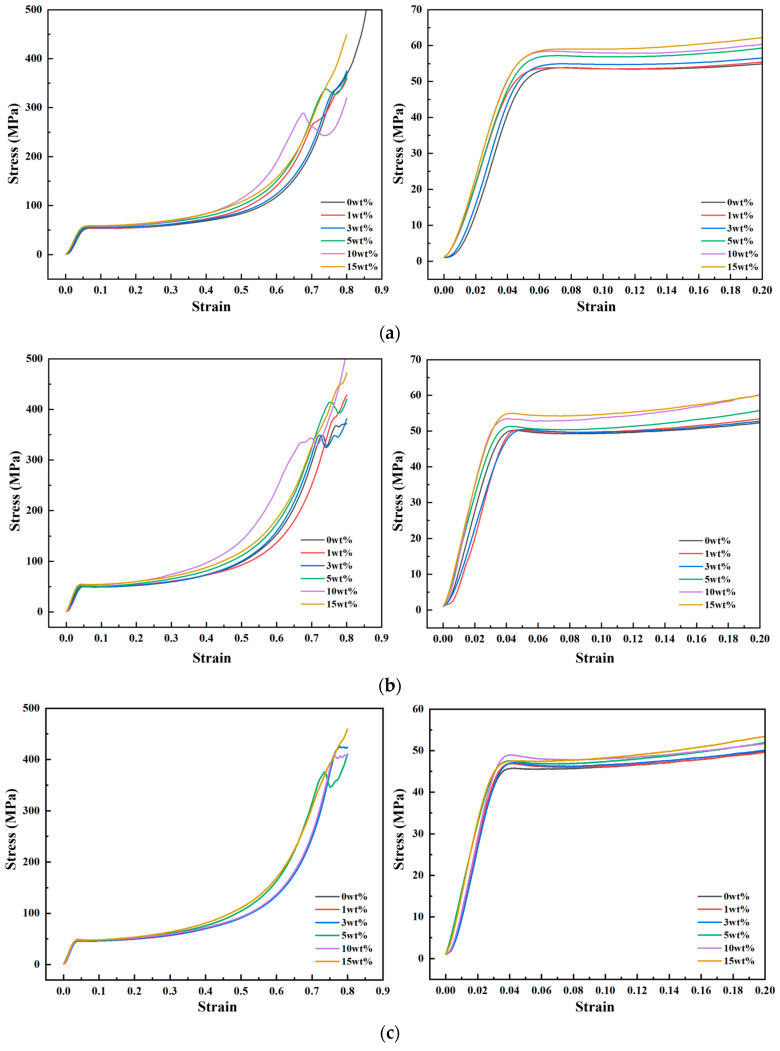
Compressive stress–strain curves of epoxy composites with various nano-Al_2_O_3_ contents tested at different strain rates: (**a**) 0.1 s^−1^, (**b**) 0.01 s^−1^, (**c**) 0.001 s^−1^.

The stress–strain curves for composites with different nano-Al_2_O_3_ contents at strain rates of 0.1 s^−1^, 0.01 s^−1^, and 0.001 s^−1^ are plotted in Figure 13. The corresponding yield strength data are summarized in Table 3.

At 0.1 s^−1^ (Figure 13a): In the small-strain range (0–0.2), the initial slope of the curve (elastic modulus) and the plateau yield stress increase monotonically with nano-Al_2_O_3_ content. The yield strength rises from ~54 MPa for neat epoxy to ~62 MPa for the 15 wt% composite (Table 3), indicating enhanced stiffness and yield resistance.

At 0.01 s^−1^ (Figure 13b): A similar strengthening trend is observed. The yield strength increases from ~50 MPa (0 wt%) to ~55 MPa (15 wt%).

At 0.001 s^−1^ (Figure 13c): While the elastic modulus still improves with filler content, the yield strength shows a non-monotonic trend. It increases up to a content of 10 wt% (~49 MPa) and then decreases at 15 wt% (~47.5 MPa), suggesting an optimal content threshold at this low strain rate.

The enhancement ratios, calculated relative to neat epoxy and listed in Table 3, further clarify these trends. At medium and high strain rates (0.01 & 0.1 s^−1^), the enhancement ratio increases monotonically with filler content, with 15 wt% providing the maximum strengthening (~9.5%). In contrast, at 0.001 s^−1^, the optimal enhancement (~7.2%) occurs at 10 wt%, beyond which agglomeration effects likely cause the enhancement ratio to drop to 4.0%.

The elastic modulus (E), calculated as the slope of the linear region (strain 0.01–0.03) using E = σ/ε, is summarized in Table 4. Across all strain rates, the modulus generally increases with nano-Al_2_O_3_ content up to 10 wt%, indicating effective reinforcement. The maximum modulus (1.75 GPa at 0.01 s^−1^, representing an 11.1% increase over neat epoxy) is consistently achieved at 10 wt%. At 15 wt%, the modulus decreases relative to the 10 wt% composite, suggesting that particle agglomeration at high content counteracts the reinforcing effect. This degradation is more pronounced at lower strain rates.

The non-monotonic influence of nano-filler content on the quasi-static compressive properties of epoxy composites, as observed in this study, resonates with findings in other particle-reinforced systems, collectively underscoring the decisive role of filler dispersion state on the final performance. For instance, Chen et al. reported in their study on sub-micron diamond particle-reinforced epoxy that the compressive strength of the composite with 5 wt% filler was even lower than that of the neat epoxy matrix, attributing this to severe particle agglomeration at high filler loading [52]. The present work extends the observation of this phenomenon to a higher filler content (15 wt%) and further reveals a coupling relationship between this “first-enhance-then-weaken” trend and the applied strain rate: the performance degradation of the 15 wt% sample, caused by agglomerates acting as defect nuclei, is most pronounced at lower quasi-static strain rates. This finding indicates that for polymer composites filled with nanoparticles, there exists a “critical content” related to processing and loading conditions. Beyond this content, agglomeration resulting from dispersion difficulties dominates the material’s behavior, thereby transforming the reinforcement phase into a source of defects. Furthermore, the nonlinear deformation exhibited in the initial stage of the stress–strain curves for all composites in this study bears similarity to the commonly observed “initial nonlinearity” in carbon fiber-reinforced epoxy (CFRP) laminates under compressive loading [53]. This commonality may originate from the viscoelastic response of the epoxy matrix itself, the closure of inherent micro-defects within the material, or early-stage micro-adjustments at the filler/matrix interface. It suggests that despite the vastly different morphologies of the reinforcement phase (continuous fibers vs. discrete particles), the contribution patterns of the matrix and the interface to the initial mechanical response of the composite possess a certain degree of universality.

**Figure 14:**
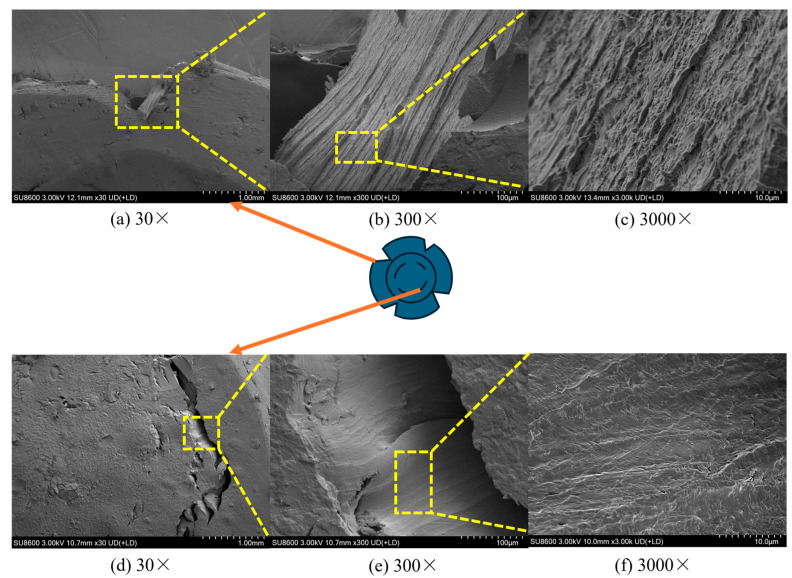
SEM images of fracture morphology for neat epoxy (0 wt%): (**a**–**c**) edge cracking zone at 30×, 300×, 3000×; (**d**–**f**) top collapse zone at 30×, 300×, 3000×.

SEM (HITACHI SU8600, Tokyo, Japan) analysis reveals the influence of nano-Al_2_O_3_ on fracture mechanisms. Neat Epoxy (0 wt%, Figure 14): The fracture surface shows features of both ductile tearing (shallow dimples in edge regions under tension) and brittle fracture (flat quasi-cleavage planes in the top collapse zone under high constraint), indicating a mixed-mode failure.

**Figure 15:**
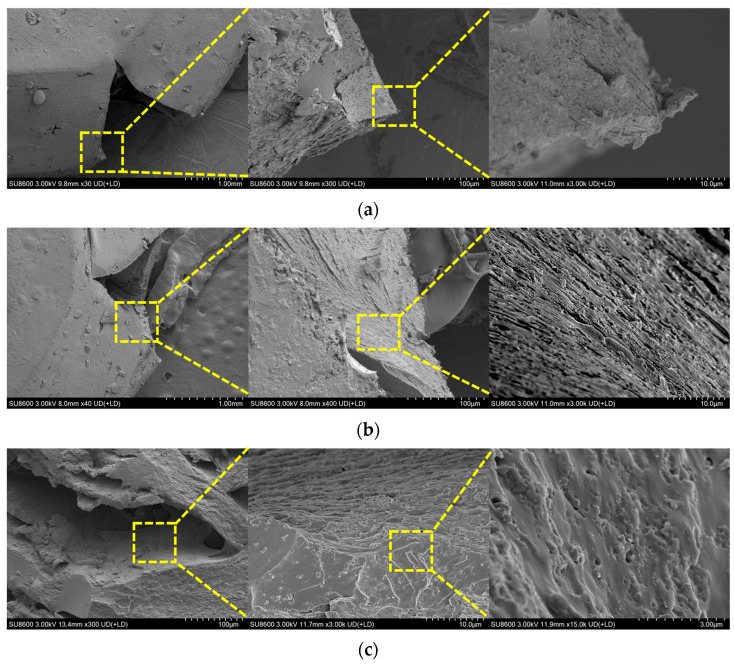
Microscopic damage morphology of epoxy composites containing different nano-Al_2_O_3_ contents after quasi-static compressive fracture: (**a**) 5 wt%; (**b**) 10 wt%; (**c**) 15 wt%.

5 wt% Composite (Figure 15a): The fracture surface is rough with numerous micro-pits and dimples, indicating a failure mode dominated by interface debonding and microvoid coalescence, offering slightly improved ductility over neat epoxy. 10 wt% Composite (Figure 15b): This composite exhibits an optimal microstructure. Particles are well-dispersed, and the fracture surface is characterized by dense, uniform dimples and layered tearing edges. Crack propagation is effectively hindered by nanoparticles, leading to extensive branching and bridging, which signifies superior ductile fracture resistance. 15 wt% Composite (Figure 15c): Severe particle agglomeration is evident. The fracture surface contains flat cleavage planes, large voids from agglomerate pull-out, and intergranular-like features. Cracks propagate rapidly along agglomeration interfaces with little deflection, indicating a reversion to brittle fracture due to excessive filler content.

In summary, under quasi-static compression, nano-Al_2_O_3_ content significantly influences the mechanical properties and failure mechanism of epoxy composites. An addition of 10 wt% provides the best compromise, optimizing particle dispersion, leading to maximum elastic modulus, and promoting ductile microvoid coalescence fracture. At 15 wt%, particle agglomeration introduces defects, reduces the modulus enhancement, and induces brittle cleavage fracture, particularly at lower strain rates. The composites also exhibit clear positive strain rate sensitivity, which is enhanced by the presence of the nanofiller.

The above micro-morphological analysis provides direct evidence for understanding the differences in macroscopic mechanical properties. The uniformly dispersed structure observed in the 10 wt% sample, along with the associated toughening mechanisms such as crack deflection and particle pull-out, is characteristic of particle-reinforced polymer composites. This aligns with the mechanisms reported in numerous studies, where particles absorb energy by hindering crack propagation and inducing interfacial debonding [52,54]. Conversely, the defects formed by agglomeration and the resulting brittle fracture observed in the 15 wt% sample are consistent with the conclusion of “performance degradation caused by agglomeration” reported by Chen et al. in a high-content diamond/epoxy system [52]. The value of this study lies in its meticulously designed filler content gradient (0, 1, 3, 5, 10, 15 wt%), which clearly and continuously demonstrates, within a single research system, the complete evolution of the microstructure from “effective toughening achieved via optimized dispersion (≤10 wt%)” to “defect aggregation induced by excessive filling (15 wt%)”. This provides intuitive experimental substantiation for the structure–property relationship between “filler content–dispersion state–mechanical performance”.

### 3.3. Analysis of Dynamic Compression Experimental Results

Dynamic compression tests on epoxy composites with four nano-Al_2_O_3_ contents (0, 5, 10, 15 wt%) were conducted using a Split Hopkinson Pressure Bar (SHPB) system at three impact pressures: 0.10, 0.15, and 0.20 MPa.

**Figure 16:**
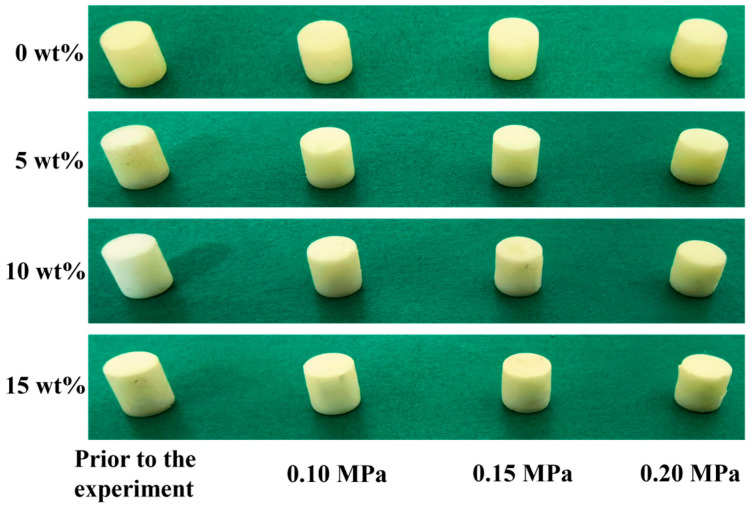
Macroscopic comparison of nano-Al_2_O_3_/epoxy composite specimens before and after dynamic compression.

Figure 16 compares the specimens before and after dynamic compression. All specimens underwent upsetting deformation characterized by radial expansion and axial shortening, with the degree of deformation increasing with impact pressure (and thus strain rate).

Dimensional measurements quantifying this deformation are summarized in Table 5. The 5 wt% composite showed the smallest dimensional change at 0.15 MPa, indicating optimal deformation resistance at this condition. The 10 wt% composite exhibited the most consistent dimensional variation across all pressures, suggesting better dimensional stability.

**Figure 17:**
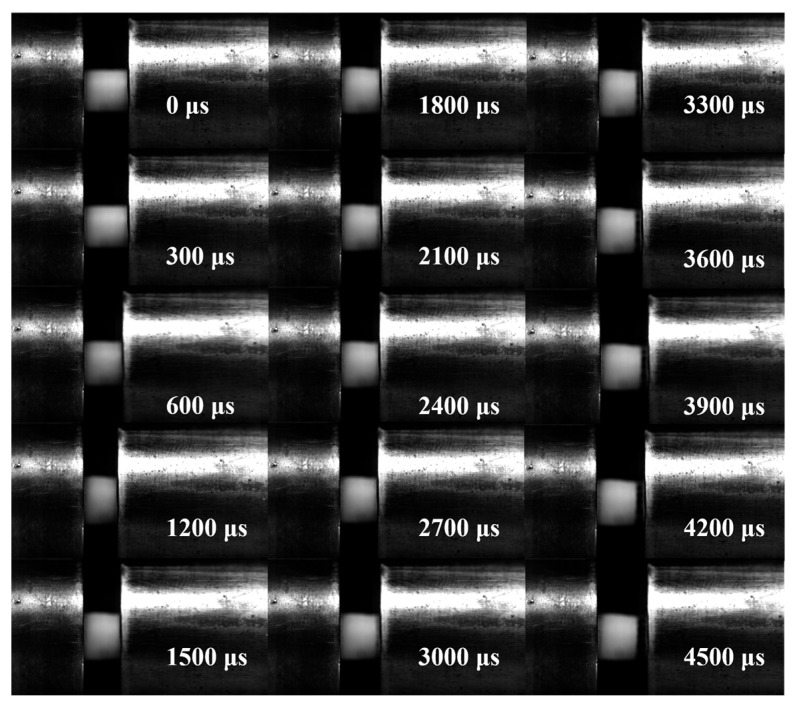
High-speed photographic sequence showing the deformation evolution of a neat epoxy (0 wt%) specimen during dynamic compression at 0.15 MPa.

Figure 17 shows the in situ morphological evolution of a neat epoxy specimen during dynamic compression at 0.15 MPa, captured by high-speed photography. The sequence clearly shows the transition from initial elastic deformation (0~600 μs) to plastic yielding with barrel-shaped bulging (1200~3000 μs), culminating in a stable, deformed profile without macroscopic fracture upon unloading (3300~4500 μs).

**Figure 18:**
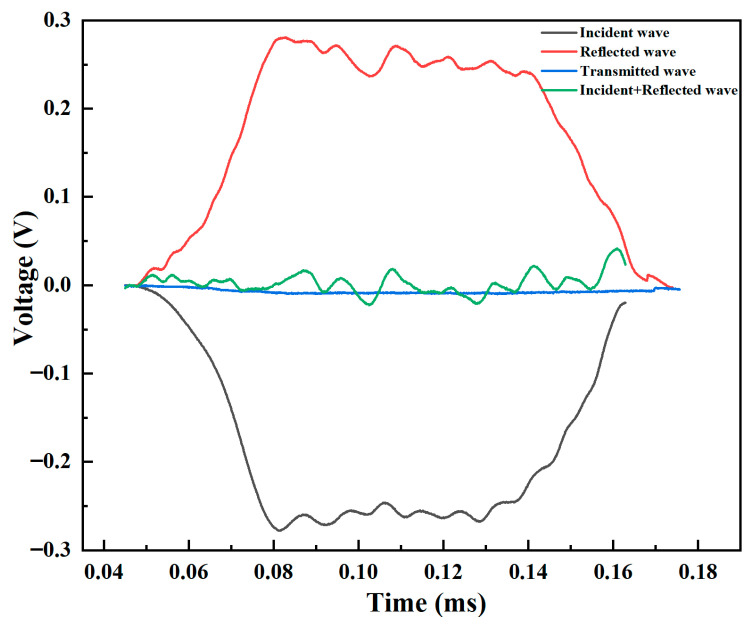
Three-wave analysis for SHPB test validation, showing incident ($\varepsilon_{i}$), reflected ($\varepsilon_{r}$), and transmitted ($\varepsilon_{t}$) waves.

The validity of the SHPB data relies on stress equilibrium within the specimen, verified by the three-wave analysis. Figure 18 shows the incident, reflected, and transmitted waves. The good agreement between $\varepsilon_{i}+\varepsilon_{r}$ and $\varepsilon_{t}$ confirms valid test conditions and reliable data.

**Figure 19:**
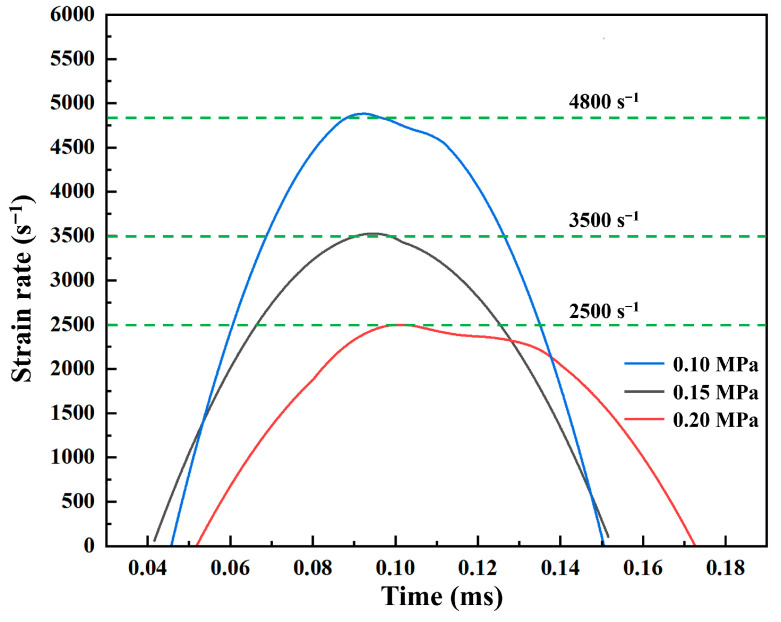
Representative engineering strain-rate versus time curves for tests conducted at impact pressures of 0.10, 0.15, and 0.20 MPa.

The corresponding strain-rate histories for the three impact pressures are shown in Figure 19, with nominal strain rates of approximately 2500 s^−1^, 3500 s^−1^, and 4800 s^−1^, respectively.

**Figure 20:**
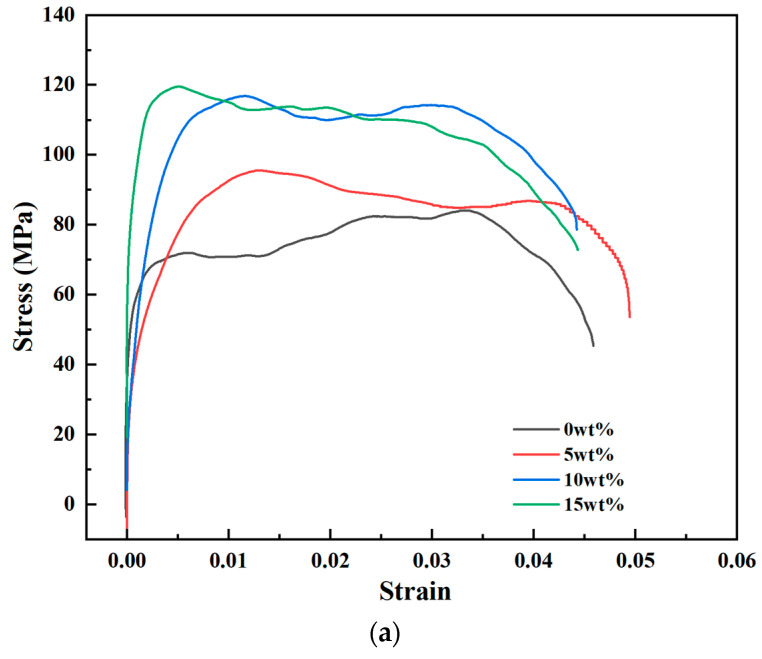

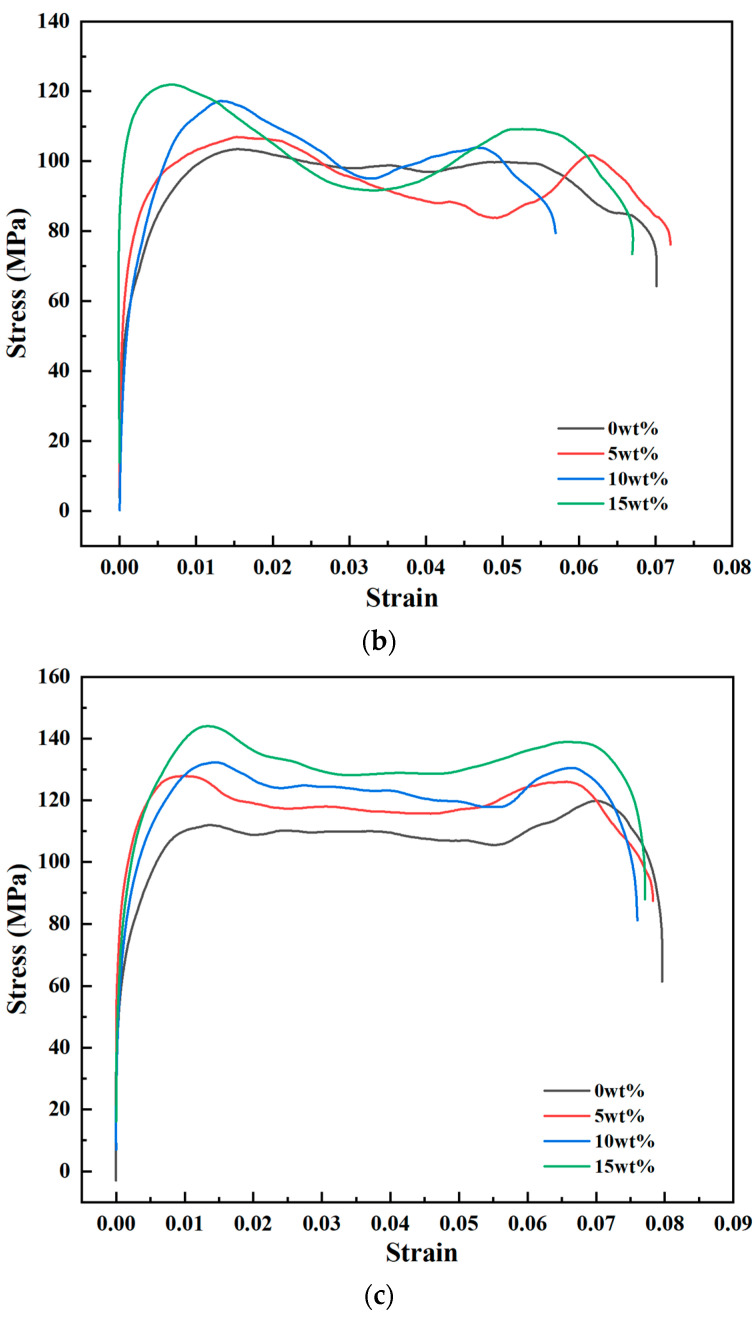
Dynamic compressive stress–strain curves of composites with different nano-Al_2_O_3_ contents at various impact pressures: (**a**) 0.10 MPa (~2500 s^−1^); (**b**) 0.15 MPa (~3500 s^−1^); (**c**) 0.20 MPa (~4800 s^−1^).

The dynamic compressive stress–strain curves for composites at each impact pressure are presented in Figure 20.

At 0.10 MPa (~2500 s^−1^, Figure 20a): The 10 wt% composite showed the optimal performance, with a high peak stress (~110 MPa) and a stable, prolonged stress plateau. The 15 wt% composite achieved a slightly higher peak stress (~120 MPa) but exhibited rapid stress decay after yielding, indicating poorer large-strain energy absorption.

At 0.15 MPa (~3500 s^−1^, Figure 20b) and 0.20 MPa (~4800 s^−1^, Figure 20c): A consistent trend was observed. While the peak stress monotonically increased with nano-Al_2_O_3_ content, the stability of the post-yield stress plateau deteriorated for the 15 wt% composite. The 10 wt% composite consistently offered the best balance of high strength and stable plastic flow.

The dynamic compressive yield stresses are summarized in Table 6. Two key trends are evident:

Filler Effect: At a constant strain rate, yield strength generally increases with nano-Al_2_O_3_ content.

Strain Rate Effect: For a given composition, yield strength increases substantially with strain rate. Notably, the enhancement ratio due to strain rate is more pronounced for the neat epoxy (~42.5% increase from 2500 to 4800 s^−1^) than for the 15 wt% composite (~20.5%), indicating that the nanofiller modulates the matrix’s intrinsic strain rate sensitivity.

The significant dynamic strengthening phenomenon observed in the nano-Al_2_O_3_/epoxy composites in this experiment is consistent with reports on similar material systems. Naik et al. reported that epoxy resin with 2 wt% and 5 wt% alumina nanoparticle additions exhibited approximately a 142% increase in compressive strength at high strain rates (~3000 s^−1^) [54]. The important extension of the present study lies in the systematic investigation of the influence of a wider range of filler contents (0–15 wt%) on this dynamic enhancement effect. The results indicate that dynamic performance does not increase monotonically with filler content. Unlike the continuous improvement observed by Naik et al. in the low-content range (≤5 wt%) [54], this study identifies 10 wt% as a critical optimal content point. At this content, the composite not only achieves high dynamic strength but also maintains a relatively stable post-peak stress plateau, indicating good energy absorption and damage tolerance. In contrast, although the 15 wt% sample may exhibit a higher peak stress, its severe post-peak softening reveals that the brittle failure mode caused by filler agglomeration is accelerated under high strain-rate loading. The discovery of this “optimal content” highlights the importance of uniform filler dispersion for maintaining structural integrity and energy absorption capacity under dynamic loading conditions; its value may even surpass that of the filler content alone. Furthermore, this strong positive strain-rate effect is not unique to nanoparticle-filled systems, as it has also been widely observed in continuous carbon fiber-reinforced epoxy (CFRP) composites [53]. This further confirms that epoxy-based composites, as a typical class of rate-sensitive materials, exhibit mechanical responses that are strongly dependent on loading history.

The modification mechanism of nano-Al_2_O_3_ in epoxy is attributed to the synergy of interface reinforcement, microstructural regulation, and strain-rate response optimization.

At an optimal content (~10 wt%), the well-dispersed, high-crystallinity lamellar α-Al_2_O_3_ particles provide extensive interfacial bonding for efficient stress transfer. They act as obstacles to crack propagation, promoting crack bifurcation and energy dissipation, leading to ductile fracture modes with stable plastic flow.

At an excessive content (15 wt%), particle agglomeration creates interfacial defects. These defects become sites for rapid crack initiation and propagation via interface debonding, resulting in brittle fracture characteristics, unstable post-yield behavior, and degraded large-strain bearing capacity—despite the higher peak stress. The overall dynamic performance is thus a synergistic result of filler content, strain rate, and dispersion state.

## 4. Conclusions

This study systematically investigated the compressive properties of nano-Al_2_O_3_-reinforced epoxy composites over a wide strain-rate range (0.001–4800 s^−1^), focusing on the effects of filler content (0–15 wt%). The main findings, supported by quantitative data, are summarized as follows:

(1) Material Characterization: The filler was confirmed as high-purity, lamellar α-Al_2_O_3_. This flake-like morphology (100–500 nm) promotes effective interfacial contact with the epoxy matrix.
(2) Quasi-Static Compression (0.001–0.1 s^−1^): The addition of nano-Al_2_O_3_ enhanced performance, but the optimal content was strain-rate dependent. At medium/high rates (0.01–0.1 s^−1^), yield strength increased monotonically with filler content, reaching a maximum increase of ~9.5% at 15 wt%. In contrast, at 0.001 s^−1^, the optimal content was 10 wt%, providing a 7.2% strength increase, beyond which agglomeration caused a reduction to 4.0%. The elastic modulus peaked at 10 wt%, with a maximum increase of 11.1%.
(3) Dynamic Compression (2500–4800 s^−1^): All composites exhibited a significant positive strain-rate effect. While strength generally increased with filler content, the 10 wt% composite delivered the optimal balance, combining high peak stress (e.g., ~133 MPa at 4800 s^−1^) with a stable post-yield plateau for better energy absorption. The 15 wt% composite, despite achieving the highest peak stress (~144 MPa at 4800 s^−1^), suffered from rapid post-peak softening.
(4) Structure–Property Relationship: Microstructural analysis directly linked performance to dispersion quality. The well-dispersed 10 wt% composite failed via ductile fracture (uniform dimples), indicating effective crack hindrance and energy dissipation. At 15 wt%, agglomeration-induced defects led to brittle cleavage, degrading performance, especially under dynamic loading.

In summary, optimal nano-Al_2_O_3_ content of ~10 wt% achieves uniform dispersion, effectively leveraging interfacial reinforcement and microstructural regulation to enhance strength, modulus, and damage tolerance across a wide strain-rate spectrum. This work provides quantitative insights and a reliable basis for designing high-performance, impact-resistant epoxy nanocomposites.

## Figures and Tables

**Table 1:** Optimization of preparation process parameters based on nano-Al_2_O_3_ content.

	Experimental Parameters	Shear Speed (r/min)	Shear Time (min)	Ultrasonic Power (W)	Ultrasonic Time (min)	Curing Temperature (°C)	Curing Time (h)
Nano-Al_2_O_3_ Content							
0 wt%, 1 wt%		300	5	250	10	25	24
3 wt%, 5 wt%		600	8	300	15	25	36
10 wt%, 15 wt%		1000	10	300	15	25	48

**Table 2:** Experimental conditions of SHPB dynamic compression tests.

No.	Nano-Al_2_O_3_ Content	Loading Pressure (MPa)	No.	Nano-Al_2_O_3_ Content	Loading Pressure (MPa)	No.	Nano-Al_2_O_3_ Content	Loading Pressure (MPa)
1	0 wt%	0.10 MPa	2	0 wt%	0.15 MPa	3	0 wt%	0.20 MPa
4	5 wt%	0.10 MPa	5	5 wt%	0.15 MPa	6	5 wt%	0.20 MPa
7	10 wt%	0.10 MPa	8	10 wt%	0.15 MPa	9	10 wt%	0.20 MPa
10	15 wt%	0.10 MPa	11	15 wt%	0.15 MPa	12	15 wt%	0.20 MPa

**Table 3:** Quasi-static yield strength and corresponding enhancement ratio of epoxy composites with different nano-Al_2_O_3_ contents.

**Nano-Al_2_O_3_ Content (wt%)**	**Yield Strength (MPa) at 0.1 s^−1^**	**Enhancement Ratio (%) at 0.1 s^−1^**	**Yield Strength (MPa) at 0.01 s^−1^**	**Enhancement Ratio (%) at 0.01 s^−1^**
0	53.76 ± 0.54	--	50.20 ± 0.50	--
1	53.82 ± 0.65	0.11	50.27 ± 0.60	0.14
3	54.92 ± 0.60	2.16	50.48 ± 0.55	0.56
5	57.14 ± 0.80	6.29	51.31 ± 0.70	2.21
10	58.46 ± 1.20	8.74	53.43 ± 0.90	6.43
15	58.87	9.51	54.98	9.52
**Nano-Al_2_O_3_ Content (wt%)**	**Yield Strength (MPa) at 0.001 s^−1^**	**Enhancement Ratio (%) at 0.001 s^−1^**		
0	45.70 ± 0.46	--		
1	46.93 ± 0.60	2.69		
3	47.05 ± 0.65	2.95		
5	47.52 ± 0.75	3.98		
10	48.97 ± 0.85	7.16		
15	47.54 ± 1.20	4.03		

Note: Yield strength data are presented as mean ± standard deviation (SD), based on measurements from *n* = 3 independent specimens. The enhancement ratio was calculated from the mean yield strength values.

**Table 4:** Elastic modulus of epoxy composites under different testing conditions.

Strain Rate (s^−1^)	Nano-Al_2_O_3_ Content (wt%)	*σ*_0.001_ (MPa)	*σ*_0.002_ (MPa)	∆*σ* (MPa)	Elastic Modulus (GPa)
0.1	0	3.40 ± 0.20	26.99 ± 1.35	23.59 ± 1.18	1.18 ± 0.06
	1	8.07 ± 0.40	35.61 ± 1.78	27.54 ± 1.38	1.38 ± 0.07
	3	4.56 ± 0.25	30.54 ± 1.53	25.97 ± 1.30	1.30 ± 0.07
	5	8.25 ± 0.45	35.98 ± 1.80	27.73 ± 1.39	1.39 ± 0.08
	10	8.64 ± 0.50	38.89 ± 1.95	30.25 ± 1.51	1.51 ± 0.08
	15	8.76 ± 0.60	38.63 ± 2.10	29.87 ± 1.65	1.49 ± 0.09
0.01	0	10.80 ± 0.65	42.21 ± 2.11	31.41 ± 1.57	1.57 ± 0.08
	1	6.38 ± 0.35	36.59 ± 1.83	30.21 ± 1.51	1.51 ± 0.08
	3	8.68 ± 0.45	37.10 ± 1.86	28.42 ± 1.42	1.42 ± 0.08
	5	15.08 ± 0.90	45.51 ± 2.28	30.44 ± 1.52	1.52 ± 0.09
	10	14.62 ± 0.85	49.52 ± 2.48	34.90 ± 1.75	1.74 ± 0.10
	15	16.28 ± 1.00	49.08 ± 2.70	32.80 ± 1.80	1.64 ± 0.11
0.001	0	9.88 ± 0.60	41.17 ± 2.06	31.29 ± 1.56	1.56 ± 0.09
	1	10.65 ± 0.65	42.29 ± 2.11	31.64 ± 1.58	1.58 ± 0.09
	3	9.51 ± 0.60	41.42 ± 2.07	31.90 ± 1.60	1.59 ± 0.10
	5	16.24 ± 1.00	44.43 ± 2.40	28.19 ± 1.55	1.41 ± 0.10
	10	11.18 ± 0.70	44.06 ± 2.20	32.88 ± 1.65	1.64 ± 0.10
	15	15.36 ± 1.10	44.72 ± 2.50	29.36 ± 1.75	1.47 ± 0.11

Note: Data are presented as mean ± standard deviation (SD), calculated from *n* = 3 independent specimens.

**Table 5:** Dimensional changes of specimens after dynamic compression.

**Nano-Al_2_O_3_ Content**	**Loading Pressure: 0.10 MPa**				**Loading Pressure: 0.15 MPa**			
	**Dia. (mm)**	**∆%**	**Ht. (mm)**	**∆%**	**Dia. (mm)**	**∆%**	**Ht. (mm)**	**∆%**
0 wt%	5.24 ± 0.05	+4.59	4.68 ± 0.05	−7.14	5.29 ± 0.06	+5.59	4.60 ± 0.05	−8.73
5 wt%	5.22 ± 0.04	+4.19	4.70 ± 0.04	−6.74	5.07 ± 0.03	+1.20	4.93 ± 0.05	−2.18
10 wt%	5.21 ± 0.03	+3.99	4.68 ± 0.04	−7.14	5.21 ± 0.04	+3.99	4.65 ± 0.05	−7.74
15 wt%	5.24 ± 0.05	+4.59	4.64 ± 0.05	−7.94	5.36 ± 0.07	+6.99	4.50 ± 0.06	−10.71
**Nano-Al_2_O_3_ Content**	**Loading Pressure: 0.20 MPa**							
	**Dia. (mm)**	**∆%**	**Ht. (mm)**	**∆%**				
0 wt%	5.45 ± 0.08	+8.78	4.48 ± 0.06	−11.11				
5 wt%	5.39 ± 0.07	+7.58	4.41 ± 0.07	−12.50				
10 wt%	5.38 ± 0.06	+7.38	4.45 ± 0.06	−11.71				
15 wt%	5.40 ± 0.07	+7.78	4.42 ± 0.07	−12.30				

Note: Dimensional data are presented as mean ± standard deviation (SD, *n* = 3). The percentage change (∆%) was calculated based on the mean dimensions relative to the initial specimen dimensions (Diameter = 5.02 mm, Height = 5.04 mm).

**Table 6:** Dynamic compressive yield stress (MPa) of the composites.

	Nano-Al_2_O_3_ Content	0 wt%	5 wt%	10 wt%	15 wt%
Strain Rate s^−1^ (Loading Pressure)					
2500 (0.10 MPa)		84.10 ± 2.10	95.57 ± 2.87	116.87 ± 3.51	119.55 ± 4.78
3500 (0.15 MPa)		103.48 ± 3.10	106.93 ± 3.21	117.19 ± 3.52	121.95 ± 4.88
4800 (0.20 MPa)		119.86 ± 3.60	127.86 ± 3.84	133.16 ± 4.00	144.02 ± 5.76

Note: Data are presented as mean ± standard deviation (SD), calculated from measurements on *n* = 3 independent specimens.

## Data Availability

The original contributions presented in this study are included in the article. Further inquiries can be directed to the corresponding author.
